# Factors associated with uptake of influenza and pertussis vaccines among pregnant women in South Australia

**DOI:** 10.1371/journal.pone.0197867

**Published:** 2018-06-14

**Authors:** Hassen Mohammed, Michelle Clarke, Ann Koehler, Maureen Watson, Helen Marshall

**Affiliations:** 1 The Robinson Research Institute, University of Adelaide, Adelaide, South Australia, Australia; 2 Adelaide Medical School, University of Adelaide, Adelaide, South Australia, Australia; 3 Vaccinology and Immunology Research Trials Unit (VIRTU), Women's and Children's Hospital, North Adelaide, South Australia, Australia; 4 The Communicable Disease Control Branch (CDCB), South Australia, Australia; 5 School of Public Health, University of Adelaide, Adelaide, South Australia, Australia; RIVM, NETHERLANDS

## Abstract

**Background:**

Maternal immunization is an effective strategy to protect pregnant women and their infants from vaccine-preventable diseases. Despite the recommendation of maternal influenza and more recently pertussis immunization in Australia, uptake of these vaccines has been suboptimal. A midwife delivered immunization program for pregnant women at the Women’s and Children’s Hospital in South Australia commenced in April 2015. Monitoring the uptake of the current funded vaccine programs for pregnant women is limited. The study aimed to estimate maternal vaccine uptake and assess factors associated with influenza and pertussis vaccine uptake among pregnant women.

**Methods:**

This prospective study was undertaken between November 2014 and July 2016 at the Women’s and Children’s Hospital. Following consent, demographic details and vaccination history for South Australian pregnant women who attended the antenatal clinic were collected. A standardised self-reported survey was completed during pregnancy with a follow up telephone interview at 8–10 weeks post-delivery.

**Results:**

205 women consented and completed the self-reported survey. Of the 180 pregnant women who completed the study, 76% and 81% received maternal influenza and pertussis vaccines respectively. The adjusted odds of women receiving maternal vaccines during pregnancy were significantly higher for women delivering after the implementation of the midwife delivered program compared with women who delivered babies prior to the program for both pertussis vaccination (AOR 21.17, 95% CI 6.14–72.95; p<0.001) and influenza vaccination (AOR 5.95, 95% CI 2.13–16.61, p<0.001). Women receiving a recommendation from a health care provider and first time mothers were significantly more likely to receive influenza vaccination during pregnancy.

**Conclusions:**

High uptake of influenza and pertussis vaccines during pregnancy can be attained with health care provider recommendation and inclusion of maternal immunization as part of standard antenatal care. A midwife delivered maternal immunization program is a promising approach to improve maternal vaccine uptake by pregnant women.

## Introduction

Pregnant women are at increased risk of morbidity and death from influenza infection during seasonal and pandemic influenza outbreaks [1–3]. This was particularly evident during the 2009 ‘H1N1’ influenza pandemic outbreak in Australia, in which the admission rate of pregnant women to an intensive care unit following infection with influenza was significantly higher compared to non-pregnant adults [4, 5]. Infants born to women affected by influenza during pregnancy are at increased risk of adverse birth outcomes such as preterm birth and low birthweight [6]. Similarily, *Bordetella pertussis* infections can also pose high risk to infants prior to their receipt of a complete primary course of pertussis immunization [7,8].

Immunization of pregnant women with influenza and pertussis has now been shown to be effective in not only protecting the mother but also the fetus /newborn via transfer of transplacental antibodies [9, 10] and through breastfeeding [11]. Maternal pertussis vaccination at least 7 days before delivery can prevent up to 91% of pertussis disease in infants under 3 months of age [12]. Similarly, influenza vaccination during pregnancy can prevent up to 91% of influenza related hospital admissions in infants under 6 months of age [13] and has been shown to reduce influenza infections in pregnant women [14]. The safety of maternal influenza and pertussis immunization is well established, with no reports of serious adverse complications to the unborn infant and pregnant women [15, 16]. Concomitant influenza and pertussis vaccination will occur in pregnancies that overlap with the influenza season, with the potential for different responses compared to separate adminsation of the vaccines in pregnant women [17]. However, a study evaluating the safety of co-administering pertussis-containing vaccine (Tdap) and influenza vaccines in pregnant women has not found an increased risk of adverse events [18].

The Australian Immunisation Handbook was updated in March 2015 to recommend pertussis-containing vaccine (Tdap) for all pregnant women during the third trimester of each pregnancy [19]. State government funded pertussis vaccination programs for pregnant women were introduced progressively between August 2014 and June 2015 in all Australian states and territories [20]. All Australian states provide pertussis vaccine for pregnant women via general practitioners and hospital antenatal clinics, local councils, community health care centres, and obstetricians. In South Australia, the funded vaccine was introduced from April 2015 and accompanied with a large state-wide promotional campaign targeting health professionals and pregnant women [20]. Influenza immunization for pregnant women has been supplied free of charge and recommended at any time during pregnancy in Australia through the National Immunization Program since 2010 [19]. Additionally, a midwife delivered maternal immunization program for influenza and pertussis vaccine was introduced in the antenatal clinic at the Women’s and Children’s Hospital in South Australia (WCH) from April 2015. This program enables registered midwives to administer maternal influenza and pertussis vaccination using a standing medication order, without the need for a prescription from a medical doctor [19].

Despite the recommendation of maternal influenza and more recently pertussis vaccinations in Australia, uptake of the recommended vaccines has historically been poor. Maternal influenza vaccine uptake in Australia has been estimated to range from about 7% to 40% [21–26]. However, these estimates are usually derived from relatively small sample studies. There are no published data on national maternal pertussis vaccine coverage in Australia. It is important to monitor and evaluate the impact of government funded pertussis vaccination programs for pregnant women and determine strategies to maximize uptake of vaccination for this population group. The primary objective of this study was to identify factors associated with the uptake of pertussis and influenza vaccines during pregnancy and to determine the uptake of influenza and pertussis vaccines among pregnant women in South Australia.

## Materials and methods

### Study population and design

This observational prospective study was undertaken between Nov-2014 and Jun-2016 at the WCH (a major tertiary maternity hospital in South Australia with an annual birth cohort of approximately 5000). Participation involved answering 26 questions about vaccination for protection against influenza and pertussis. A total of 300 pregnant women were approached and invited to participate in this research study. Participation in the survey was voluntary. A research nurse/medical officer discussed the study with the participants prior to obtaining written informed consent

### Eligibility criteria

Pregnant women were eligible to participate if they were aged 18 years or over at the time of enrolment and had sufficient understanding of the English language. Pregnant women were eligible to partake in this study regardless of their gestational stage or expected delivery date.

### Data collection instrument

A standardised self-report questionnaire was designed to collect socio-demographic details and information on awareness and uptake of the recommended maternal influenza and pertussis immunizations among pregnant women. A follow up telephone interview were conducted 8–10 weeks post-delivery. Participants were classified as ‘lost to follow- up’ and omitted from the analysis, if incorrect contact details were provided, if they refused further participation, or did not answer six phone call attempts at different times and on different days. The follow up telephone interview included questions to confirm whether they received influenza and/or pertussis vaccination during their pregnancy, a date and location, and if not during pregnancy, whether they had received influenza or pertussis vaccine post birth of their baby (See supplement). Delivery date of the woman was used to compare maternal influenza and pertussis immunization coverage prior to and following the implementation of the midwife delivered maternal immunization program. The midwife vaccine delivery program equipped midwives with knowledge and skills to engage with pregnant women on the topic of maternal immunizations and administer pertussis and influenza immunizations to pregnant women [27].

### Statistical analysis

The sample size was calculated assuming 55% of pertussis vaccine uptake during pregnancy based on a self-reported survey (FluMum cohort study) [28] in 2015 collected over a three month period at the Women’s and Children’s Hospital in South Australia. For this study, an expected sample size of 200 participants enabled the uptake of pertussis vaccination amongst pregnant women to be estimated and to determine predictors of maternal influenza and pertussis vaccination uptake with a ±5% precision at a 95% confidence level.

Survey data were analysed using STATA Version 14. Descriptive analysis such as proportions for categorical variables and mean (median) for continuous variables were calculated. Chi squared tests (χ^2^) were used to determine any crude association between categorical variables. Results were considered to indicate statistical significance, if a two-tailed p-value was less than 0.05. Univariate and multivariable logistic regression models were used to estimate unadjusted (ORs) and adjusted odds ratios (AORs) to identify variables related to the uptake of maternal influenza and pertussis vaccines.

### Human research ethics approval

The study protocol was approved by the Women’s and Children’s Health Network Human Research Ethics Committee (HREC/14/WCHN/3).

## Results

### Participant’s characteristics

**Fig 1:**
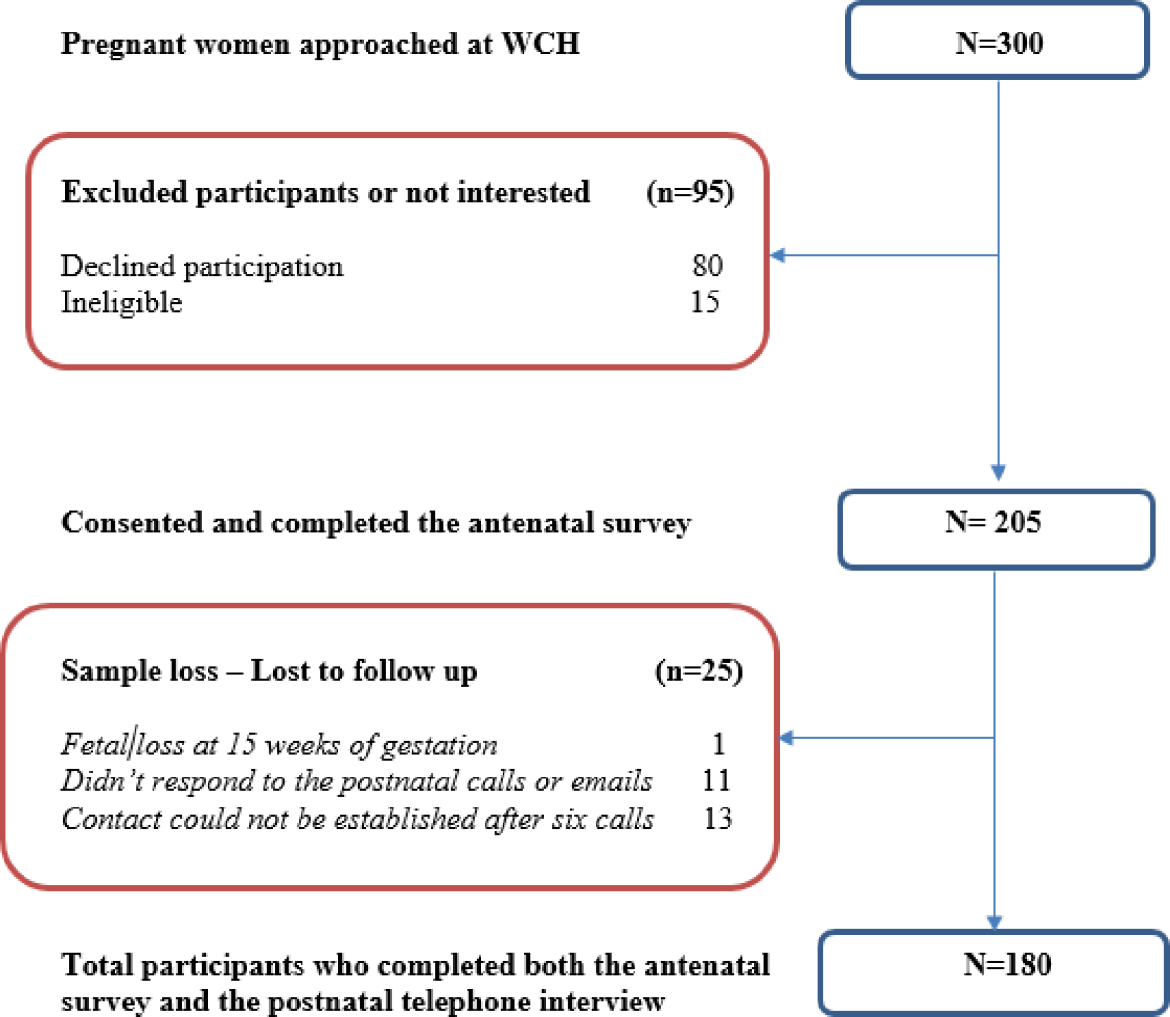
Recruitment flowchart.

Of 300 pregnant women approached at the WCH, 205 women consented and completed the antenatal survey questionnaire. Of the 205 participants, 24 were lost to follow up for the postnatal interview and one participant was excluded from the study because of fetal death during the pregnancy. Overall, 180 (88%) of the enrolled participants completed both the antenatal survey and the postnatal follow-up telephone call questionnaire (Fig 1). Data analysis was performed based on the 180 participants who completed both portions of the study.

The median age of participants was 31.1 years (range 21–43 years old), similar to the median age (30.6 years) of South Australian pregnant women reported by Australian Bureau of Statistics (ABS) in 2014 [29]. The majority of the pregnant women who participated in this study were born in Australia (74%) (Table 1). These sample characteristics are similar to South Australian for pregnant women according to the 2013 Pregnancy outcome SA report [30]. No indigenous women particpated in this study. A total of 82 (46%) of the women were first time mothers, while 54% of the participants were multiparous (Table 1).

**Table 1:** Baseline characteristics of the study population.

		Study population (n = 180)	
Characteristics	Level	Total number	Percentage
**Maternal Age**	18–31	96	53
	32–41	84	47
**Born in Australia**	Yes	116	74
	No	42	26
**Parity**	Primiparous	82	46
	Multiparous	98	54
**Pregnancy trimester (at the time of enrolment)**	1^st^	17	9
	2^nd^	62	34
	3^rd^	101	56

NB: Country of birth missing data, n = 22.

### Uptake of the recommended maternal vaccines

#### Pertussis

Almost all the participants (167/180, 93%) had heard of ‘pertussis’, regardless of their immunization status. Of the 180 participants, 66% (n = 119) were aware of the recommendation of pertussis vaccination during the 3rd trimester and 46% (n = 83) were aware that they could receive a pertussis vaccination shortly after delivery if the vaccine was not given during pregnancy. Overall, 82% (148/180) of the participants received pertussis vaccination; 81% (n = 145/180) during pregnancy and 2% (n = 3/180) post-delivery of their baby. Overall, 63% (92/145) of the women reported receiving the vaccine during pregnancy from a midwife during their antenatal visit at WCH, 32% (47/145) from a general practitioner (GP), 3% (4/145) from an occupational immunization provider and 1% from a community health center. Women who had heard of the availability of maternal pertussis vaccination prior to study participation had almost 8 times higher odds of receiving the vaccine during pregnancy (OR 7.8, CI 3.3–18.3; p<0.001) (Table 2). Almost all infants 97% (175/180) were vaccinated with the routine diphtheria, tetanus and acellular pertussis (DTaP) vaccines (scheduled at 6–8 weeks of age).

**Table 2:** Factors potentially associated with pertussis vaccine uptake during pregnancy.

			Univariate binomial regression			Multivariable logistic regression		
Variable	Level	Received maternal pertussis vaccine n (%)	Odds ratio (OR)	95% CI	p-value^b^	Adjusted odds ratio (AOR)^a^	95% CI	p-value
**Maternal age category**	21–31	85/96 (89%)	1.00			1.00		
	32–43	60/82 (73%)	0.35	0.18–0.78	0.010	0.39	0.130–1.11	0.078
**Country of birth**	Australia	102/116(88%)	1.00					
	Other	32/42(76%)	0.30	0.28–2.14	0.124			
**Parity**	Primiparous	70/82 (85%)	1.00					
	Multiparous	75/98 (77%)	0.53	0.24–1.18	0.116			
**Awareness of maternal pertussis recommendation**	No	37/64 (63%)	1.00			1.00		
	Yes	108/117(82%)	7.78	3.31–18.2	<0.001	4.43	1.61–12.23	0.009
**A midwife delivered maternal immunization program**	Prior	5/25 (20%)	1.00			1.00		
	Post introduction	140/155(90%)	31.73	10.25–98.27	<0.001	21.17	6.14–72.95	<0.001

^a^Adjusted odds ratio comparing odds of receiving pertussis vaccine during pregnancy if offered, controlling for other variables.

^b^Only univariate associations with p value <0.1 were included in the multivariable logistic regression.

#### Influenza

Overall, 80% (144/180) of the women who participated in this study received influenza vaccination; 76% (n = 136) of the participants received influenza vaccination during pregnancy and 5% (n = 8) received the vaccine post-delivery. Overall, 38% of the women reported receiving the vaccine during pregnancy from a general practitioner (GP), 37% from a midwife at WCH, 8% from occupational immunization provider, 1% from a community health center and 16% of the women failed to report where they have received the vaccine. Of the 180 study participants, 82% of them were aware that influenza vaccine is recommended during pregnancy, 67% were aware that they could receive influenza vaccine at any stage of their pregnancy and 65% of the women had discussed maternal influenza vaccination with their health care providers (HCPs). Pregnant women who had received a recommendation from their HCP had 3 times greater odds of receiving maternal influenza vaccination than women who had not received a recommendation (Table 3). Of the 130 women who received influenza vaccination during pregnancy (6 participants did not report their dates of vaccination), the majority (62%, 81/130) received the influenza vaccine in April (n = 47) or May (n = 34). A further 45 women (35%) received the vaccine during the influenza season which is typically between May to October in South Australia, with very few women (3%) being vaccinated between January and March. It should also be noted that influenza vaccine has generally not been available between January and March prior to release of the new seasonal vaccine. Among the most common reasons women cited for not receiving the vaccine during pregnancy were lack of recommendations from their HCPs (28%, n = 14) (Table 4).

**Table 3:** Factors potentially associated with influenza vaccine uptake during pregnancy.

			Univariate binomial regression			Multivariable binomial regression		
Variable	Level	Received maternal influenza vaccine n (%)	Unadjusted odds ratio (OR)	95% CI	p-value^b^	Adjusted odds ratio (AOR)^a^	95% CI	p-value
**Maternal age category**	21–31	81/96 (84%)	1.00			1.00		
	32–43	55/83 (66%)	0.36	0.17–0.74	0.005	0.40	0.17–0.92	0.031
**Country of birth**	Australia	91/116 (78%)	1.00					
	Other	32/42 (76%)	0.50	0.16–1.57	0.763			
**Parity**	Primiparous	69/82 (84%)	1.00					
	Multiparous	67/97 (71%)	0.42	0.20–0.87	0.021	0.43	0.19–0.99	0.048
**Provider recommendation received**	No	40/64 (63%)	1.00			1.00		
	Yes	96/115 (83%)	3.03	1.49–6.14	0.001	2.81	1.19–6.68	0.002
**A midwife delivered maternal immunization program**	Prior	8/25 (32%)	1.00			1.00		
	Post introduction	128/155(83%)	8.00	3.06–20.91	<0.001	5.95	2.13–16.61	<0.001

^a^ Adjusted odds ratio comparing odds of receiving influenza vaccine during pregnancy if offered, controlling for other variables.

^b^ Only univariate associations with p value <0.1 were included in the multivariable logistic regression

**Table 4:** Reasons cited for not receiving maternal influenza vaccination.

Reasons cited for NOT receiving the influenza vaccination during pregnancy	Number (n)	Percentage %
It was not suggested/recommended to me	14	28%
Prior experience of an adverse reaction after being vaccinated	8	16%
I did not know that pregnant women should be vaccinated	8	16%
I was unsure of the benefits or effectiveness of the vaccine	5	10%
Never had time to receive the vaccine	3	6%
Received the vaccine earlier this year	3	6%
I was not pregnant during the flu season	3	6%
Prefer natural immunity	2	4%
Flu vaccine exacerbates my Asthma	2	4%
Prefer to receive the vaccine after the baby is born	2	4%

NB. Women were allowed to report >1 reason.

### Maternal vaccine uptakes pre-post introduction of a midwife delivered immunization program at WCH in SA

#### Pertussis

**Fig 2:**
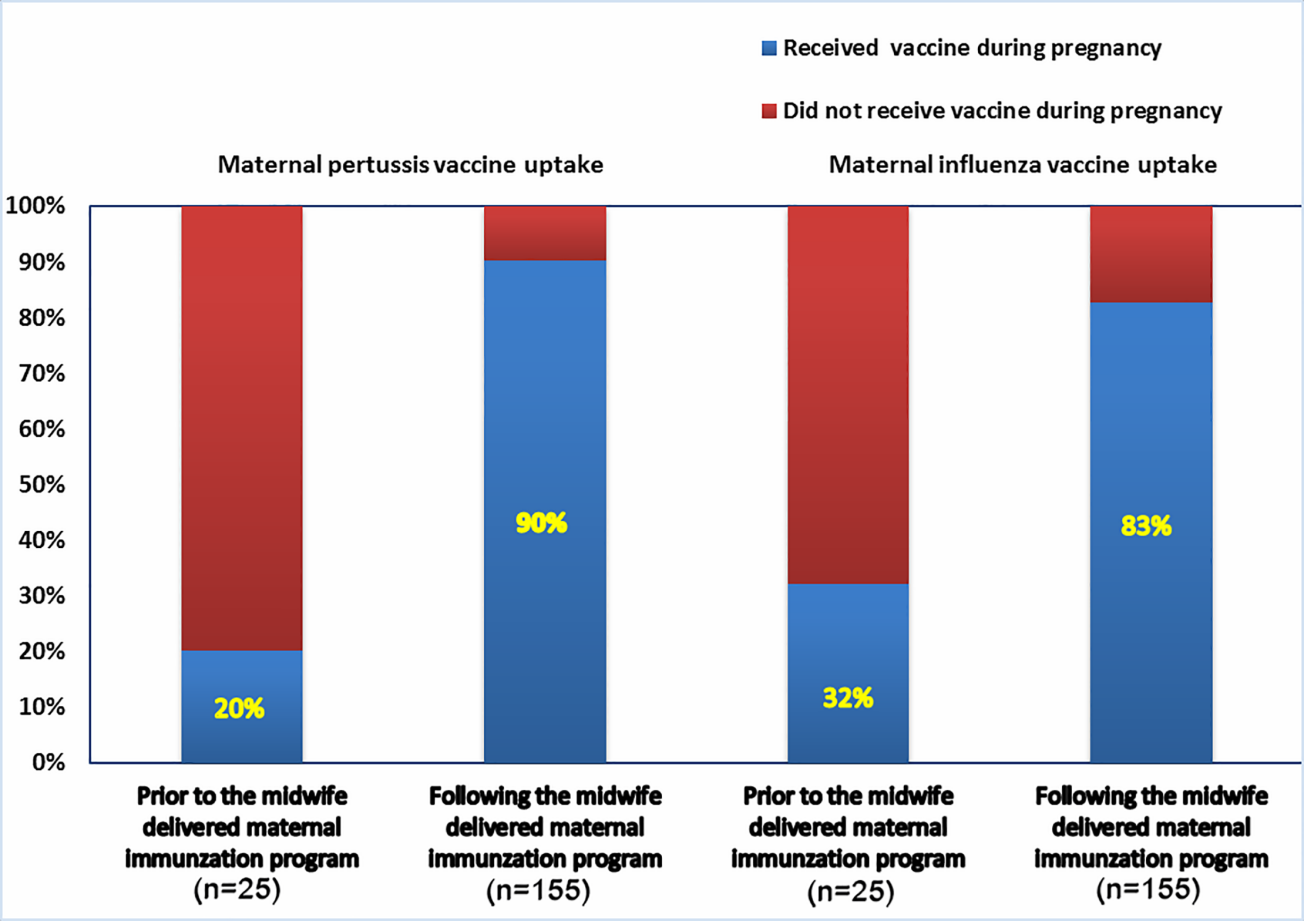
Maternal receipt of pertussis and influenza vaccine pre-and post-implementation of a midwife delivered maternal immunization program at the WCH in South Australia.

The proportion of women who received pertussis vaccine during pregnancy following the introduction of the midwife delivered vaccination program for pregnant women was significantly higher (140/155, 90%) compared with women who delivered prior to the introduction of the program (5/25, 20%; p <0.001) (Fig 2). The univariate odds of women receiving maternal pertussis vaccine following the implementation of the government funded pertussis program and midwife delivered maternal immunization program in the antenatal clinic of the WCH was almost 32 times higher than women who delivered babies prior to the program (OR 31.73, CI 10.24–98.27; p<0.001) (Table 2).

#### Influenza

Women who had received pertussis vaccine during pregnancy were also more likely to have both been recommended the influenza vaccine during their pregnancy (90% vs 66%; p<0.001) and been immunized against influenza during pregnancy (92% vs 49%, p<0.001) compared to pregnant women who had not received pertussis vaccine. The univariate odds of women receiving influenza vaccine following the implementation of the midwife program was 8 times higher than women who have given birth prior to the program (OR 8.0, CI 3.06–20.9; p<0.001) (Table 3).

### Association between socio-demographic factors and maternal vaccination rates

#### Maternal age

The median age of participating women was 31 years. Maternal age older than 31 years was associated with lower uptake of maternal influenza and pertussis immunizations. The odds of older women (32–43 years) receiving maternal influenza vaccine was less than half that of younger women (OR 0.36; CI 0.17–0.74; p = 0.005). In multivariable logistic regression analysis, maternal age remained a strong predictor for the uptake of influenza vaccine during pregnancy (AOR 0.40; CI 0.17–0.92 p = 0.031) (Table 3). Similarly, the odds of older women receiving maternal pertussis vaccine was less than half that of younger women (OR 0.35; CI 0.18–0.78; p = 0.010). However, after adjusting for all independent variables, the association between maternal age and pertussis vaccine uptake during pregnancy was no longer statistically significant (AOR 0.39 CI 0.130–1.11; p = 0.078) (Table 2).

#### Country of birth

The proportion of women vaccinated against influenza and pertussis during pregnancy was not statistically significantly different between women who were born in Australia and women born overseas (78% vs 76%; p = 0.763) (88% vs 76%; p = 0.124) (Tables 2 and 3).

#### Parity

In the univariate analysis, the odds of multiparous women having received maternal influenza vaccine was less than half that of first time mothers (OR 0.42 CI 0.20–0.87; p = 0.022). In multivariate analysis, the odds of mothers with previous children receiving maternal influenza vaccine remained less than half that of women with no previous children (AOR 0.43; 95% CI 0.19–0.99 p = 0.048) (Table 3). However, for pertussis vaccine uptake, whilst the odds of multiparous women receiving the vaccine was lower compared to first time mothers in a univariate analysis, this was not statistically significant (OR 0.53 CI 0.24–1.18; p = 0.116) (Table 2).

## Discussion

Our results showed high uptake of pertussis (81%) and influenza (76%) vaccines during pregnancy. The higher uptake of pertussis vaccine during pregnancy compared to influenza vaccine in our study could be because most women perceive influenza as a disease affecting the mother, whereas they see pertussis as a threat to the infant and thus relatively more risky [31]. Uptake of maternal pertussis vaccine by women who delivered at the WCH prior to the government funded immunization programs was 20%, which significantly improved to 90% following the introduction of a midwife delivered and government funded pertussis program. Similarly, the uptake of influenza vaccine during pregnancy has improved from 32% to 83% following the implementation of a midwife delivered immunization program. National coverage for maternal pertussis vaccination programs in other countries is limited to local surveys of vaccine coverage, for example an estimated coverage of over 60% was reported in the UK in 2016 [32], and 51% of women delivering during March 2014 in Wisconsin [33] while uptake of 51–67% was reported in Argentina in 2014 [34].

The rise in vaccination rates could also be attributed to introduction of free pertussis vaccine for all pregnant women in South Australia in March 2015 [15]. The higher uptake of pertussis compared to influenza vaccine suggests pertussis vaccine uptake is driving influenza vaccine uptake in pregnant women as the vaccines are co administered. Our results demonstrate that the provision of maternal pertussis and influenza vaccination by midwives at the place of antenatal service was an independent strong predictor of vaccination uptake during pregnancy. It is a relatively low cost intervention, which has produced a significant increase on vaccine uptake. Pregnant women view midwives as a trusted source of health information [35]. A previous study suggested that administering maternal immunizations into standard antenatal care through midwives could improve immunization uptake among pregnant women [36].

Our study demonstrated that receiving a recommendation from a HCP was a strong predictor for receipt of maternal influenza vaccine. Women who had not received influenza vaccine during their pregnancy were less likely to have been offered influenza vaccines. About one-quarter of the women who had not received the vaccines reported they had not received a recommendation to have influenza vaccine during pregnancy. This suggest there is room for improvement for HCPs in discussing maternal vaccinations with pregnant women. Several other studies suggested that a recommendation from a HCP is the most significant factor in improving vaccination uptake during pregnancy [27, 37, 38].

Previously identified factors associated with poor uptake of vaccines in pregnancy include lack of perceived benefit by pregnant women [22], concern about the safety of maternal vaccination [39], lack of awareness of vaccine recommendation during pregnancy [40, 41] and expectant mother’s attitudes toward immunization during pregnancy [42, 43]. In this study, maternal age and parity were associated with uptake of influenza vaccines during pregnancy. Multiparous women were less likely to be vaccinated against the influenza during pregnancy compared to first time mothers. A similar finding has been reported from a previous study in South Australia [44]. Multiparous women are more likely to attend fewer antenatal visits than first time mothers [45]. They also tend to have lower emotional attachment to their unborn baby [46] and have been found to think less about the health of their fetus than first time mothers [47]. This may describe why women who had been pregnant before were less likely to be immunized against influenza vaccine during pregnancy. However, our findings have shown that there is no influence of parity on pertussis vaccine uptake during pregnancy

Our study also demonstrated that older women were less likely to be vaccinated against influenza during pregnancy. A previous study also suggested that older women are less likely to seek antenatal health care [48]. This could explain why older women were less likely to be vaccinated against influenza during pregnancy. Our study findings indicate the need for tailored and targeted interventions for older multiparous women in maternal influenza vaccination campaigns. However, a study conducted in the Netherlands has found that influenza vaccine uptake during pregnancy was higher among older and multiparous pregnant women which is in contrast to our study findings [49]. Our study also indicates that there is no influence of age on pertussis vaccination coverage, which is in contradiction with previous studies [44, 50]. Hence, further research is needed to explore if older multiparous women are more or less likely to receive maternal influenza or pertussis vaccine compared with young first time mothers.

The primary strength of our study sample is the inclusion of pregnant women prior to and following the implementation of a midwife delivered pertussis immunization programs for pregnant women. This enabled us to compare the antenatal vaccination uptake rates prior to and following the introduction of midwife delivered maternal immunization program. This study has also examined the intention of pregnant women to receive the recommended vaccines during or post pregnancy and uptake of these vaccines was verified by follow up telephone interview with the mothers after delivery.

This study was subject to some limitations. The participants in this study were recruited through a public hospital antenatal clinic. Thus, the study findings may not be a representative of the overall population of pregnant women in South Australia. Our relatively small sample size could also be a limitation to this study. Results from the vaccination coverage before the government funded immunization programs are based on a very small sample size and this could be a potential limitation of the study. The study sample also excluded non-English-speaking women and no indigenous women participated in the study therefore our findings may not be representative of culturally and linguistically diverse (CALD) and Aboriginal and Torres Strait Islander women. Vaccine uptake among women who reported they had received influenza and pertussis vaccination during their pregnancy was not verified through audit of medical records. Our study has also a potential selection bias, as women who are more accepting of vaccination may have been more likely to agree to participate in the survey. Another limitation of the study is that the questionnaire did not capture primary language, ethnicity, household income, educational level, working situation and marital status, which may also be important variables in assessing factors related to vaccine uptake during pregnancy.

## Conclusions

High uptake of influenza and pertussis vaccines during pregnancy can be attained with health care provider recommendation and inclusion of maternal immunization as part of standard antenatal care. A midwife delivered maternal immunization program is a promising approach to improve maternal vaccine uptakes by pregnant women. Additional studies are needed to monitor and evaluate the impact of government funded pertussis programs for pregnant women to ensure optimum protection for pregnant women and their infants.

## Supporting information

S1 File Maternal vaccination survey. (DOCX)

S2 File Dataset. (XLSX)
