# Supplementary figures and images for: Factors associated with uptake of influenza and pertussis vaccines among pregnant women in South Australia
Source: PLoS One. 2018 Jun 14;13(6):e0197867. doi: 10.1371/journal.pone.0197867 (PMC6002099; doi:10.1371/journal.pone.0197867)

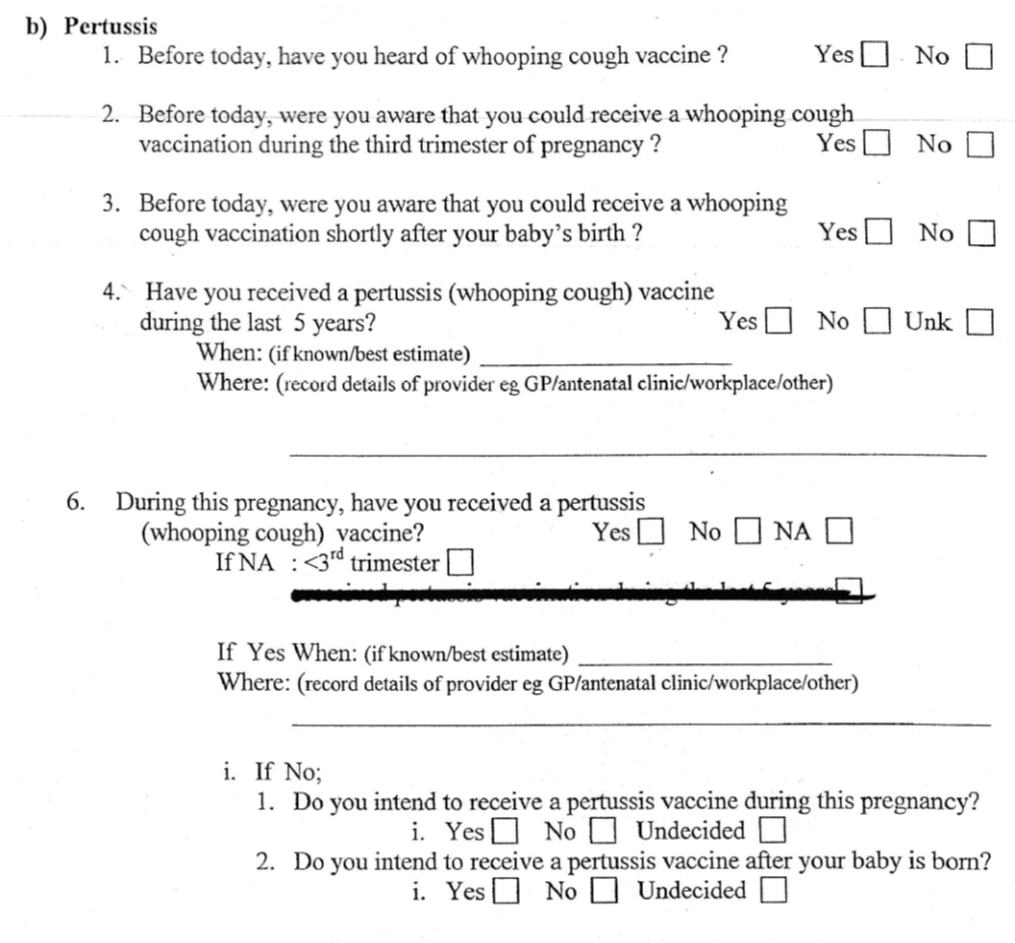


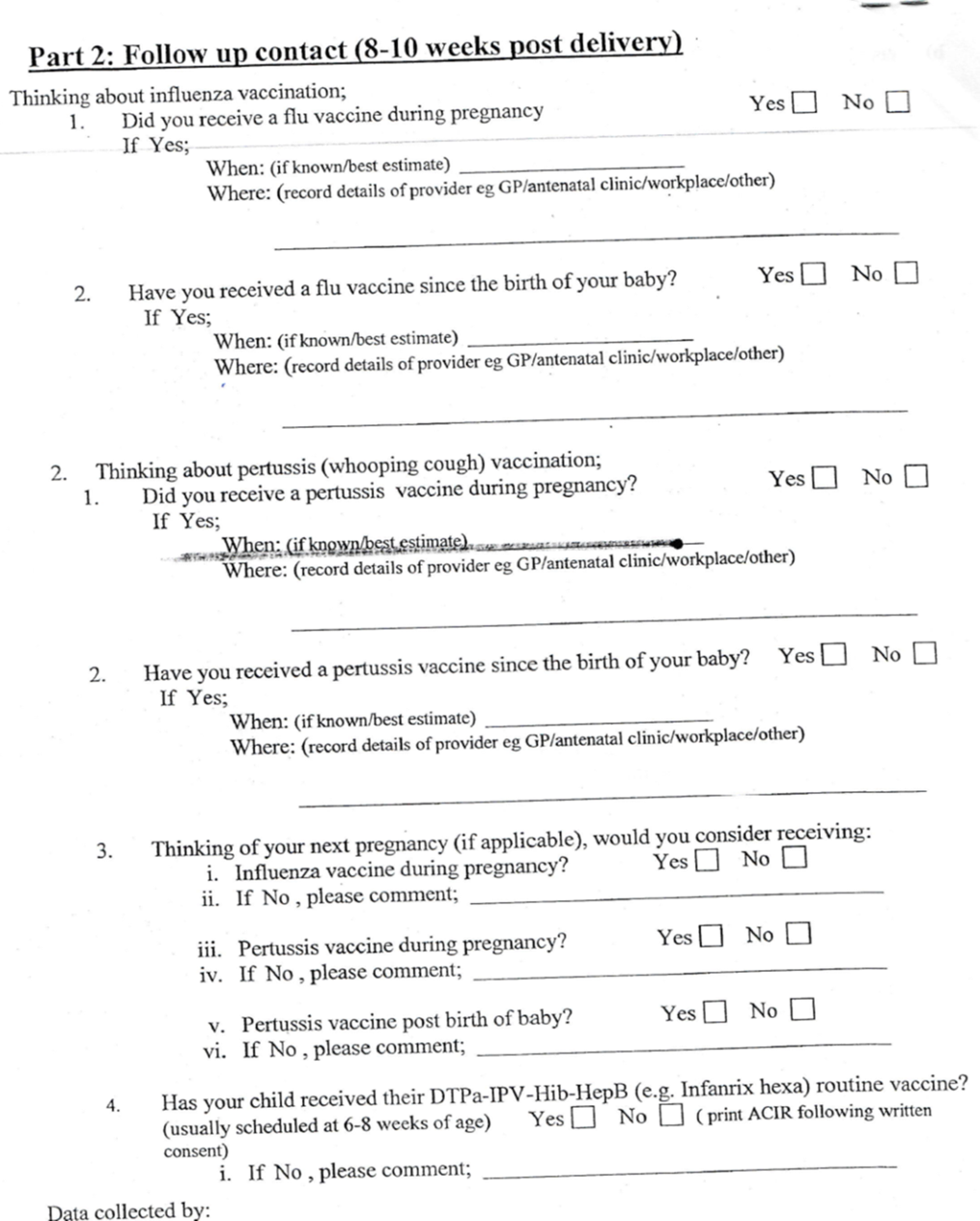

Supplement: S1 File — (DOCX) [file pone.0197867.s001.docx]
